# Building Polyacryronitrile Fiber/Epoxy Resin (PANER) Interleaving Film to Strengthen Flexural and Compressive Performances of Laminated CFRP Composites

**DOI:** 10.3390/nano15201576

**Published:** 2025-10-16

**Authors:** Sidra Ashfaq, Jiaxin He, Yanan Lyu, Fei Cheng, Xiang Yuan, Xueling Liang, Shuying Shi, Evgeny Lomakin, Daria Bondarchuk, Rasuljon Tojiyev, Hao Liu, Xiaozhi Hu, Xi Chen

**Affiliations:** 1 Shock and Vibration of Engineering Materials and Structures Key Lab of Sichuan Province, School of Materials and Chemistry, Southwest University of Science and Technology, No. 59 Middle Section of Qinglong Avenue, Fu Cheng District, Mianyang 621010, China; sidriashfaqkhan@gmail.com (S.A.); hejiaxin2023@163.com (J.H.); 18382884069@163.com (X.Y.); zjh20001106@163.com (X.L.); wjssyshi@163.com (S.S.); 2 Institute of Automation, Chinese Academy of Sciences, Beijing 100190, China; lvyanan2018@ia.ac.cn (Y.L.); xi.chen@ia.ac.cn (X.C.); 3 School of Artificial Intelligence, University of Chinese Academy of Sciences, Beijing 100101, China; 4 Department of Plasticity, Lomonosov Moscow State University, Moscow 119991, Russia; evlomakin@yandex.ru (E.L.); bondarchuk.da@gmail.com (D.B.); 5 Department of Technological Machinery and Equipment, Fergana State Technical University, Fergana 150100, Uzbekistan; yiyi_iiid@163.com; 6 School of Aerospace Engineering, Tsinghua University, No. 1 Tsinghua Yuan, Haidian District, Beijing 100084, China; drhaoliu@tsinghua.edu.cn; 7 School of Mechanical Engineering, University of Western Australia, Perth 6009, Australia; xiaozhi.hu@uwa.edu.au

**Keywords:** Carbon Fiber Reinforced Polymer (CFRP), polyacryronitrile (PAN) fibers, fiber bridging, flexural and compressive strength, interlaminar toughening

## Abstract

Carbon fiber-reinforced polymer (CFRP) composites have excellent mechanical properties, but their performance is hampered by delamination caused by weak interfacial bonding and resin-rich region (RRR). This research has proposed an interleaving film to improve interlaminar structure and mechanical properties by adding polyacrylonitrile (PAN) fiber into the epoxy interlayer of the CFRP laminates. The PAN fiber/epoxy resin (PANER) interleaving film could be prepared, which was beneficial to hinder crack initiation paths and improve the load transfer. Flexural and compression performance testing results showed optimum performance was obtained when 2 wt.% PAN fiber was added, and an increment of 28.6% was obtained in the flexural strength and 11.7% increment in compressive strength. The damaged energy absorption was improved up to 21.4% and 11.3% for the flexural and compressive properties, respectively. The overall thickness increments in the interlayer with PANER interleaving film were approximately 4–9 μm. X-Ray micro-computed tomography and scanning electron microscopy observations exhibited the potential of PAN fiber in the reduction of RRR, resulting in modes replacement from delamination-dominant failure to crossing-multi-layer failure. In all, PANER interleaving film at the interlayer has been confirmed to be an effective approach to produce a simple reinforcement technology for FRP laminates.

## 1. Introduction

Carbon fiber-reinforced polymer (CFRP) is a versatile and high-performance composite material consisting of carbon fibers embedded within a polymer matrix, exhibiting an outstanding strength-to-weight ratio. The anisotropic nature of CFRP allows tailored designs where strength in specific directions is crucial, making it ideal for various civil engineering applications. CFRP offers superior mechanical properties, including high modulus, high stiffness, high tensile strength, fatigue resistance, and low density, while remaining lightweight [1,2,3]. Due to its durability, corrosion resistance, and structural flexibility, CFRP has become a critical material across industries like aerospace, automobile, sports equipment, ship, and many other manufacturing sectors [4,5].

Despite its excellent mechanical properties, CFRP is a laminated structure in which the bonding between the CF and the resin matrix is a key factor. The weak adhesive bonding leads to delamination in the laminated structure when it suffers any external load, which ultimately results in the failure of the laminates. Delamination is mainly caused by the poor interfacial adhesive bonding and resin enrichment in the weak region, which could be called the resin-rich region (RRR) during the preparation or the curing process of the composite. The RRR is easy to crack because of the brittleness of epoxy resin; those generated cracks will propagate along the bonding adhesive region between the CF and the resin matrix layer, which is called the interfacial transition region (ITR), causing the internal delamination [3,6,7,8].

Different methods have been proposed to overcome the aforementioned issues, like the generation and propagation of cracks, which ultimately lead to the delamination failure of the composite materials. Many researchers have used various methods such as Z-pinning, stitching, tufting, and 3D weaving [9,10,11,12,13,14,15,16], but more complex processes need to be conducted. Even if these methods can improve interfacial performance, some important properties are likely to be compromised. For example, z-pinning can enhance through-the-thickness strength and damage resistance, but at the same time cause the fiber damage or misalignment and stress concentration, which may lead to the overall performance failure of fiber-reinforced polymer (FRP) composites [17,18]. Moreover, these methods require more precise control and advanced machinery to avoid causing unnecessary defects. Additionally, using these complex processes to produce CFRP cannot match the requirements and demands of the lightweight and high-performance materials nowadays in different fields.

Therefore, the more sustainable method used to develop the high-performance FRP composites was the direct introduction of the reinforcing fibers or the additives into the adhesive interlayer [19,20]. Zhou et al. utilized a hybrid interleaving strategy combining hierarchical carbon nanotubes and short carbon fiber, achieving a 125% improvement in fracture toughness. An areal density of 1.0 mg/cm^2^ of mixing enhancer was applied [21]. Kumar et al. introduced nanographene to improve the toughness properties of epoxy and the delamination mode of its reinforced composite. As a result, mode I and mode II delamination resistance increased by about three times and 2.5 times, respectively, and about a 100% increment was observed in the crack propagation resistance [22]. Yuan et al. interleaved the CFRP interface ply with ultra-thin short aramid fiber veils to construct the fiber bridging; about 16.9% and 19.8% increments were obtained in the modulus and bulk flexural strength of CFRP [23]. Ou et al. reported the use of carbon nanotube veils (about 100–300 nm in thickness) between the CF layers in a laminated structure, and results indicated a remarkable increment in the Mode I interlaminar fracture toughness of up to 107% [24]. Oh et al. reinforced unidirectional CF/epoxy laminates also by incorporating the polyethylene-terephthalate-based thermoplastic veils that were modified by spray-coating them with carbon nanofibers and coupling agents. The multiscale reinforcement approach provided synergies that enhanced Mode I and Mode II interlaminar fracture toughness by up to 105% and 611%, respectively [25]. In recent studies, researchers have reinforced FRP and CFRP composites with different interleaving materials. The research revealed that polyamide veils, PEEK films, and other thermoplastics, to a large extent, enhanced fracture toughness and delamination resistance. Furthermore, Fine Glass (FG) veils improved the bending stiffness, and the impact resistance was also improved when an elastomer layer was added to the hybrid laminates [26,27,28,29,30].

All these methods have shown a great relation between the interlayer structure and the performance of the FRP composites. Strong bonding between the adhesive layer and the CF layers would have a synergistic effect on the strength, as well as the properties, of the composites without compromising the in-plane characteristics of the composites. Although interlayer reinforcement has been widely investigated using different above-mentioned reinforcing materials, PAN fiber has been rarely used as a reinforcing fiber to improve the interlaminar structure. It is a synthetic polymer fiber made up of polyacrylonitrile, a precursor material for the production of high-performance and high-modulus carbon fibers. It possesses good tensile strength and elastic modulus, along with excellent fatigue and corrosion resistance, as well as low density [31,32]. It also has good thermal and chemical resistance and a hydrophobic nature (low moisture absorption) because of the presence of nitrile groups (–C≡N) that repel water. These fibers are widely used as thermal insulation, chemical-resistant filters, and concrete additives, as well as playing a key role in the primary components of aircraft structures like wings, fuselage, empennage, and rocket fairings due to their role in carbon fiber manufacturing [33,34,35,36].

**Figure 1:**
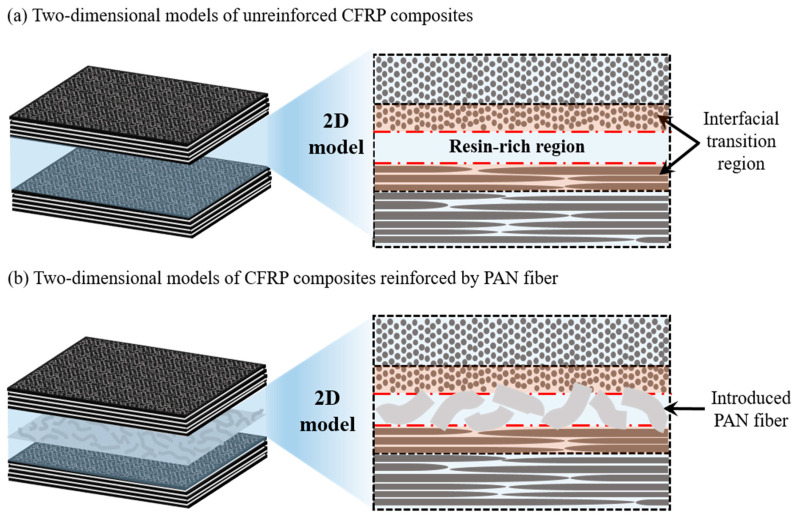
Two-dimensional models: (**a**) unreinforced CFRP composites; (**b**) CFRP composites reinforced by PAN fiber.

PANER interleaving film could be formed by using PAN fiber to mix with epoxy between the CF layers in the laminated CFRP structure, which is able to improve the interlaminar structure and performance of the CFRP composites, as illustrated in Figure 1. PAN exhibits a good interfacial adhesion with thermoset matrices like epoxy, which can improve the load transfer at the interlaminar interface. By forming an interlocking bridging between the resin matrix and the CF layers, the PAN fiber can tightly hold the CF plies together, thereby reducing the risk of crack generation and propagation in the weak regions like RRR and ITR. This mechanism could be helpful to suppress delamination under the external load and contribute to the development of high-performance composites. PAN fiber incorporated into the interlayer as a reinforcing material has shown a good impact on the mechanical properties, as well as the structural integrity of the CFRP composites. Three-point bending tests (3PB) and compression tests were carried out to study the flexural and compressive strength of the PAN-reinforced CFRP composites. Optical microscopy and scanning electron microscopy were used to understand the interlaminar distribution of the incorporated PAN fibers within the CFRP laminates after damage. X-ray micro-computed tomography was employed to investigate the crack propagation modes within the laminated structure and to evaluate the fracture failure of the composites. A simple and effective technique was developed to reinforce CFRP composites using PANER interleaving film, and its reinforcing mechanism was investigated.

## 2. Preparation and Characterization of CFRP Composites

### 2.1. Raw Materials

The main starting materials used in the fabrication of CFRP composites reinforced by PAN fiber were CF fabric, PAN fiber, epoxy resin, and hardener. All details about the raw materials, including their physical properties and manufacturer, are listed below in Table 1.

### 2.2. Preparation of CFRP Composites Reinforced by PAN Fiber

**Figure 2:**
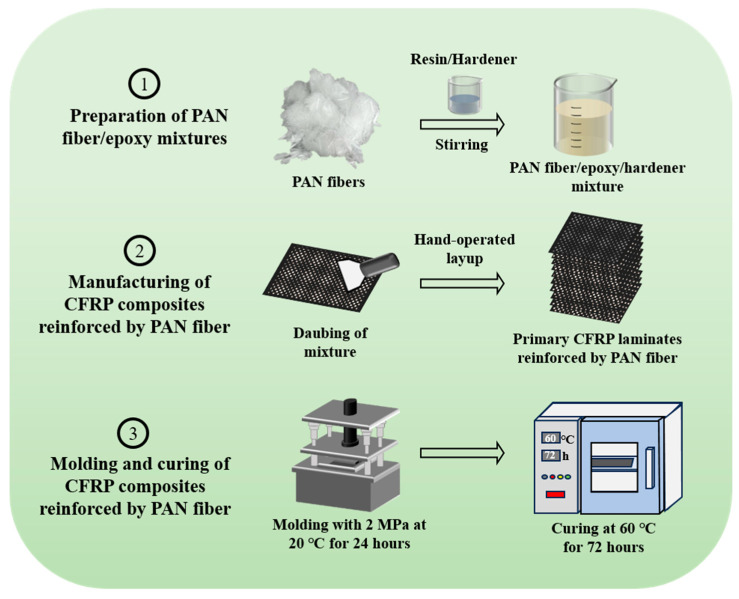
Schematic diagram of preparation and manufacturing of CFRP composites.

Fabrication of CFRP composites with different masses of PAN fibers was carried out in different ways, as shown in Figure 2.

(1) PAN/epoxy mixtures were first processed without the addition of hardener. In this step, the continuous PAN fibers were finely cut into short, discontinuous fibers, and then mixed with epoxy resin in various masses. The PAN ratio was varied as 1 wt.%, 2 wt.%, 3 wt.%, and 4 wt.%. By thoroughly stirring the PAN fibers into epoxy resin, a uniform and homogeneously viscous PAN/epoxy mixture was prepared.
(2) After preparing the mixture, the process was followed by calculating and adding the hardener at a ratio of 1:5 relative to the epoxy resin content in the PAN/epoxy mixtures. Then, PAN/epoxy/hardener mixtures with varying masses of PAN fibers were daubed onto the carbon fiber fabric to design the primary CFRP laminated structure reinforced by PAN fibers.
(3) The final CFRP composite structure was obtained after compression molding and curing. The prepared primary-laminated CFRP was placed under compression molding at 2 MPa for 24 h and then placed into the dry curing chamber at 60 °C for 72 h to obtain finally cured laminated CFRP composites. The detailed parameters of final CFRP composites reinforced by PAN fiber are presented in Table 2.

### 2.3. Tests and Characterizations of CFRP Composites Reinforced by PAN Fiber

The PAN fiber was scanned using a scanning electron microscopy (SEM, ZEISS Gemini 300, Carl Zeiss GmbH, Oberkochen, Germany) at a voltage of 3 kV in order to reveal its microstructure on the PAN surface. The PAN fiber was examined using a fourier transform infrared spectroscopy (FT-IR, Frontier, PerkinElmer, Waltham, MA, USA) to acquire spectral characteristics. Thermal stability and heat adsorption/release of PAN fiber were conducted using thermogravimetric-differential scanning calorimetry (TG-DSC, STA8000, PerkinElmer, Waltham, MA, USA). The specimen was heated at a rate of 10 °C/min under atmospheric nitrogen from room temperature to 800 °C.

The flexural performance of CFRP composites was investigated by three-point bending (3PB) testing using the MTS CMT4104 universal testing machine with a 10 kN load cell (Jinan MTS Test Technology Co., Ltd., Shandong, China). Standard test specimens of CFRP composite were prepared with the corresponding dimensions of 80 mm × 13 mm. The testing span width of supporting two points was set to be 57.6 mm, as specified by ASTM D7264 [37]. For each group, five specimens containing different PAN mass contents were fabricated. The loading nose moved downwards at a constant rate of 2 mm/min by applying force on the specimens until a sudden drop in load was observed, indicating specimen failure.

To examine the compressive strength of the specimens, compression tests were carried out using a 100 kN universal testing machine. In accordance with ASTM D6641 [38], CFRP composite specimens were prepared in a standard configuration with dimensions of 140 mm in length and 13 mm in width. A combined compression loading (CLC) fixture was used to apply compressive loads through both end and shear loading mechanisms, minimizing the risk of specimen buckling. Five specimens from each group were tested, and a uniform crosshead displacement rate of 1 mm/min was maintained during the tests. Compressive force was applied until specimen failure, and the compressive strength and modulus were determined from the resulting stress–strain curves.

The interlaminar structures of the plain and reinforced CFRP composites were examined using an optical microscope (OM, WUMO WMJ-9590, Shanghai WUMO Optical Instrument Co., Ltd., Shanghai, China), and the interlaminar distribution of PAN fiber at the interlayer was observed and captured through a built-in WUMOview3.7 WM-3000C software. To observe the microstructure of the damaged surface of the specimen after 3PB tests and analyze the reinforcing mechanism of the residual PAN fiber at the interlayer, the scanning electron microscopy (SEM, ZEISS Gemini 300, Carl Zeiss GmbH, Oberkochen, Germany) was again employed at a voltage of 3 kV.

A high-resolution open-tube X-ray microscope (XRM-CT) system (NanoVoxel-3502E, Sanying Precision Instruments Co., Ltd., Tianjin, China) was used to scan the interlayer of the CFRP composites after 3PB tests for understanding the internal crack propagation. The equipment worked at a scanning resolution of 1 μm, a constant voltage of 60.0 kV, and a current of 40.0 μA.

## 3. Results and Discussions

### 3.1. Microstructure, Chemical Composition, and Thermal Stability of PAN Fiber

**Figure 3:**
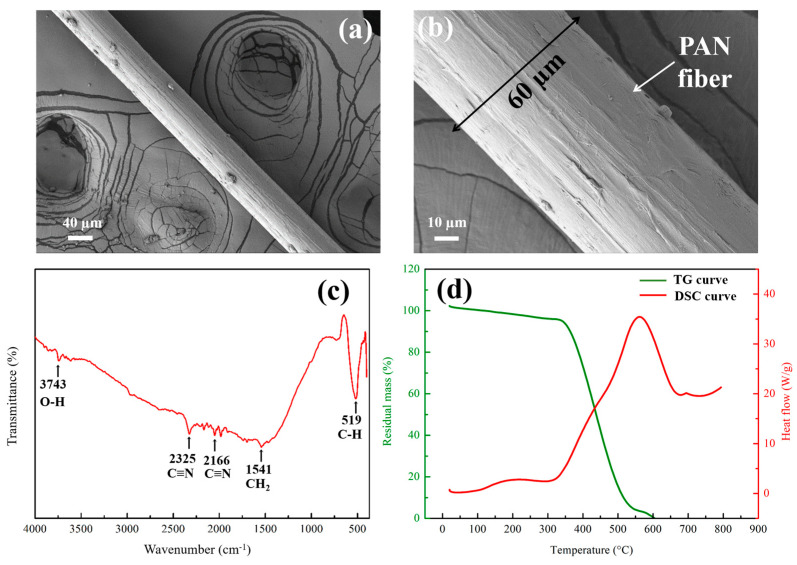
Microstructure, chemical composition, and thermal stability of PAN fiber: (**a**,**b**) SEM images; (**c**) FT-IR spectrum; (**d**) TG-DSC curves.

The microstructure of PAN fiber was exhibited in Figure 3a,b, where it was found that the PAN fiber had a uniform and smooth structure, and the diameter was approximately 60 μm. Figure 3c exhibited the FT-IR spectrum of PAN fiber, and the specific absorption peaks were noted at the positions of 3743, 2325, 2166, 1541, and 519 cm^−1^. The highest absorption peak at 3743 cm^−1^ was attributed to stretching mode of O-H owing to the hydroxyl group or a little moisture. The absorption bands related to the stretching vibration of C≡N at 2325 cm^−1^ and C=N at 2166 cm^−1^, stating the typical nitrile structure of PAN. The maxima at 1541 cm^−1^ were associated with bending vibration of C-H in CH_2_ and absorption in 519 cm^−1^ was attributed to the stretching vibration of C-H group.

TG-DSC curves of PAN fiber were exhibited in Figure 3d, where the endothermic peak in the DSC curve within the 100–500 °C was due to the thermal degradation (cyclization) of the nitrile groups (C≡N) in the PAN polymer backbone. In TG curve, a little weight loss was observed from 100–350 °C, mostly due to removal of absorbed water and initial decomposition of side groups. A sharp mass loss was observed in the TG curve around 350–500 °C corresponded to the main degradation of PAN fiber associated with cyclization and dehydrogenation of nitrile groups, releasing volatile byproducts (such as hydrogen cyanide and acrylonitrile). The endothermic peak centered at 400 °C in the DSC curve corresponded to a maximum degradation temperature, where major thermal decomposition of PAN fiber took place. Overall, the thermal stability of PAN fiber was good.

### 3.2. Interlayer Fiber Distributed Structure of Laminated CFRP Composites

**Figure 4:**
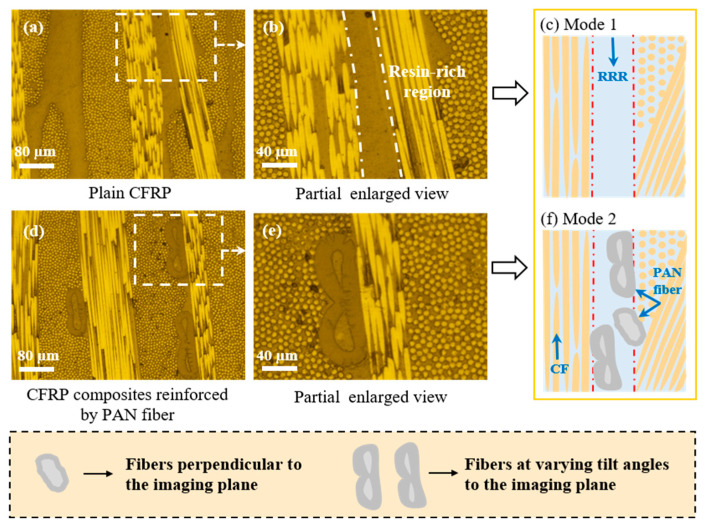
Photographs of interlayer structure of CFRP composites: (**a**–**c**) Plain CFRP composites; (**d**–**f**) CFRP composites reinforced by PAN fiber.

In Figure 4, both the unreinforced and reinforced interlayer fiber distributions were displayed. The incorporation of PAN fiber into the interlayer played a crucial role in improving the structural integrity and performance of CFRP laminates. An obvious RRR could be observed in the unreinforced composite in Figure 4a–c, and its thickness reached around 80 μm, as it was clear that the brittle RRR is susceptible to crack initiation and propagation throughout the interlayer [39] and was not good for manufacturing a high-performance composite. While it was observed in the representative reinforced interlayer structure that the original RRR could not be found, as shown in Figure 4d–f, the reason was that PAN fiber embedded into the epoxy interlayer and, hence, reduced the volume fraction of epoxy in RRR by hindering the unnecessary accumulation of resin. It could be observed that the added PAN fiber did not increase the thickness of the interlayer. In Figure 4f, the clear distribution patterns of PAN within the interlayer were analyzed, showing different fiber directions and distinct structural features of PAN fiber, which also contributed to formation of multi-directional fiber bridging at the interlayer.

### 3.3. Flexural and Compressive Properties of CFRP Composites After Tests

**Figure 5:**
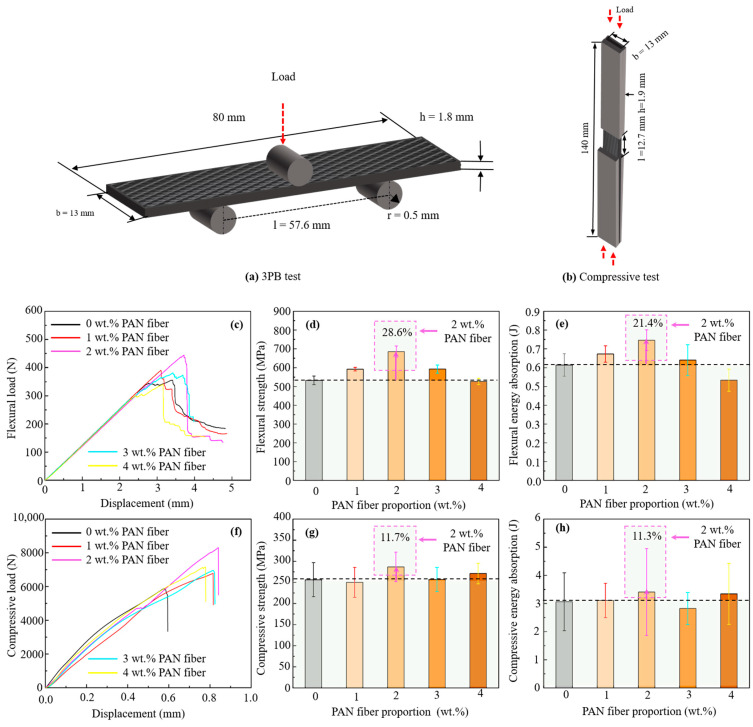
Related results of CFRP composites reinforced by PAN fiber with various conditions after flexural and compression testing: (**a**,**b**) schematic drawing of CFRP composites for 3PB and compression tests; (**c**,**f**) typical load-displacement curves; (**d**,**g**) average flexural and compressive strength; (**e**,**h**) flexural and compressive energy absorption.

The flexural and compressive tests of CFRP composites were conducted to evaluate the influence of PAN fiber on interlayer reinforcement; the related results are depicted in Figure 5 and Table 3. Compared to the plain CFRP composites, CFRP composites reinforced by PAN fiber exhibited a variation in both flexural and compressive properties. As shown in Figure 5c,f, the load-displacement curves for plain CFRP composites exhibited lower peaks, indicating a small load-carrying capacity compared to reinforced CFRP composites, while CFRP composites with 2 wt.% PAN fiber had the maximum load-carrying capacity (P_max_) under bending and compression load. As Figure 5c,f displayed, load-displacement curves could be divided into linear and dropping zones for both the flexural and compression tests. After the load was applied, when specimens reached the maximum load-carrying peak, crack generation occurred, which ultimately caused the structural deformation. Further, 2 wt.% of PAN fiber contributed to achieving the highest load peak point and the maximum displacement compared to plain CFRP composites. Additionally, the CFRP laminates with PAN fiber basically had an improvement in the flexural and compressive strength as illustrated in Figure 5d,g. Among them, the plain CFRP composites had 532.76 MPa in flexural and 256.46 MPa in compressive strength, respectively. The specimen with 2 wt.% of PAN fiber still yielded the best strength, including 685.61 MPa in flexural strength and 286.66 MPa in compressive strength, exhibiting 28.6% and 11.7% improvements, respectively, over the plain CFRP composites. Moreover, its energy absorption values calculated at the peak load for the 3PB tests and compression tests were also improved up to 21.4% and 11.3%, respectively, as demonstrated in Figure 5e,h. The positive influence of PAN fiber may be attributed to its multi-directional distribution at the interlayer, which could fill the adhesive layer to toughen the epoxy resin and avoid RRR, and also create the disordered fiber bridges. These behaviors were of benefit to high-performance development of laminated CFRP composites. It was observed that 2 wt.% of PAN fiber provided optimum improvement in flexural and compressive performances as the additive fibers were uniformly dispersed in the resin matrix. This uniform dispersion of fibers allowed the effective load transfer from the resin matrix to the reinforcement and resulted in strong interfacial bonding. However, at higher mass proportions of PAN fiber, these fibers might have started to agglomerate, and their dispersion within the polymer matrix became uneven, which led to weak interfacial bonding. These agglomerates acted as the stress concentrators, which in turn reduced the load-carrying capacity of CFRP composites, and hence caused the decline in the strength and overall performance of CFRP composites under external loading conditions.

### 3.4. Fractured Surface Analysis

**Figure 6:**
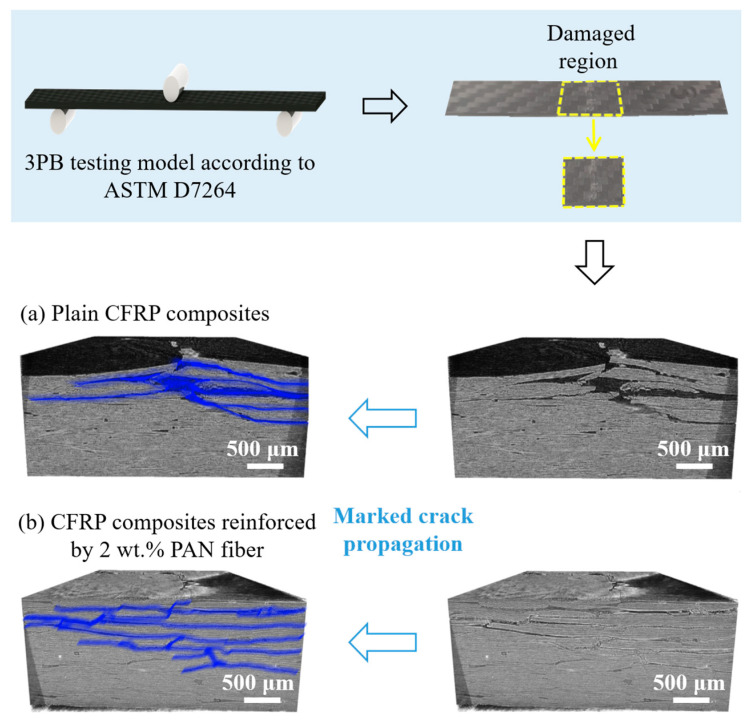
Three-dimensional XRM-CT images of internal fractured surfaces: (**a**) plain CFRP composites; (**b**) CFRP composites reinforced by PAN fiber.

XRM-CT was employed to analyze the fractured path and the propagation of the generated cracks after 3PB testing. The area marked in blue represented the direction of the crack propagation in Figure 6. It could be observed that the main propagation direction of cracks was parallel to the fiber layer, as in Figure 6a for the plain CFRP composites. When the cracks were generated in the RRR, they propagated along the direction of the epoxy adhesive, and the delamination failure of the composites was caused [40]. It indicated that the toughness in the unreinforced CFRP composites was not sufficient to withstand the stress situations. On the contrary, the composites reinforced by PAN showed relatively different behavior under the bending force, even though the crack propagation path parallel to the fiber layer could be found, but those cracks were crossing the multi-layers to form the longer crack propagation perpendicular to the fiber layer, as shown in Figure 6b. It could be considered that the clear delamination-dominated failure of the plain CFRP composites was shifted to the multi-layer damage [41] of the specimen with PAN fiber, and these introduced PAN fibers hindered the propagation of the cracks within the interlayer.

### 3.5. Microstructure Analysis of Damaged Surfaces of CFRP After 3PB

**Figure 7:**
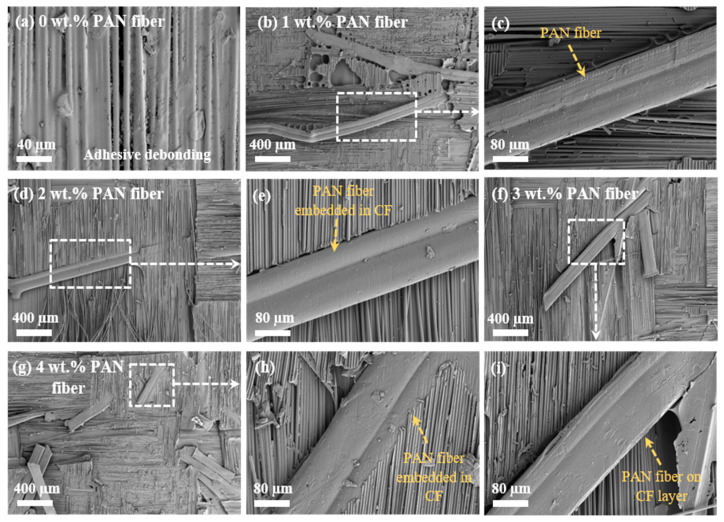
SEM images of damaged surfaces of CFRP composites reinforced by different mass proportions of PAN fiber after 3PB testing: (**a**) plain CFRP composites; (**b**–**i**) CFRP composites reinforced by PAN fiber.

Figure 7 illustrates damaged surface SEM images of CFRP composites with various masses of PAN fiber after 3PB tests. To justify the reinforcing mechanism of PAN on CFRP composites, it was necessary to find the fiber bridging at the interlayer or the ITR. Figure 7a displayed the damaged interface of plain CFRP composite; the dominant debonding could be observed between the CF layers, confirming the delamination failure of the laminated composite. In Figure 7b–i, original cylindrical PAN fibers were compressed into a flat fiber. It could be also observed that those flat PAN fibers were pulled off from the PANER interleaving film and still kept the construction of fiber bridging between the CF layers, forming the delamination-free structure. The good adhesion between CF layers and PAN fibers resulted in the strong interface formation and reduced the risk of crack generation and propagation within the ITR, forming a stiff and robust laminated structure.

### 3.6. Contribution of PANER Interleaving Film in Improving CFRP Composites

**Figure 8:**
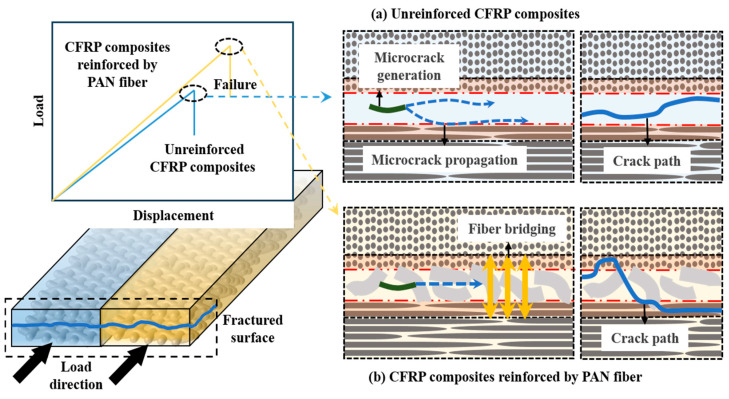
Reinforcing mechanism of PANER interleaving film: (**a**) unreinforced CFRP composites; (**b**) CFRP composites reinforced by PAN fiber.

Issues that undermined interlayer integrity in laminated CFRP composites were large resin-rich regions (RRR) and weak interlocking interlaminar transition regions (ITR). The brittle RRR was weak and susceptible to generation of micro-cracks under certain external loads. Once initiated, the micro-cracks would propagate along the ITR or the epoxy layer, and it was easy to cause delamination to progress under the continuous load [40], as shown in Figure 8a. Both the unreinforced and CFRP composites with PAN fiber exhibited totally different behavior. This failure behavior was greatly changed by the introduction of PANER interleaving film at the interlayer. The PAN fibers optimized the amount of RRR and stiffened the adhesive layer, resulting in a more homogeneous load transfer and reductions of local stress concentrations, due to which early cracking would have been generated.

PAN fibers were able to move into the epoxy layer and flow between ply-to-ply carbon fiber fabrics during the curing procedure. The fiber-bridging network was formed in many directions, which could grasp the effect in different directions of the adjacent plies and limited the propagation of cracks along the RRR and ITR, as depicted in Figure 8b. More additional fracture energies were required to break the CF out or to pull off the PAN fibers embedded in the composites. In addition, the increasing amounts of PAN fiber should be noticed; more PAN fibers would lead to an interlayer thickness that is not a favorable result in reinforcing CFRP composites. As the thickness increment in the interlayer might affect the mechanical properties of the laminated structures, therefore, in this study, a thin PANER interleaving film was introduced between the CF layers, which had a thickness increment of about 4–9 μm.

## 4. Conclusions

PAN fibers were introduced into the interlayer of the CFRP composites to form the PANER interleaving film for improving the RRR and ITR of the CFRP composites in this study. Flexural and compressive strength of the CFRP composites was compared to confirm the synergistic effect of PAN fiber. Some highlights were summarized as follows.

(1) PANER interleaving film was successfully prepared at the interlayer of CFRP composite; the fiber bridging behavior was also constructed between CF layers, which could improve the mechanical interlocking of CFRP laminates to hinder the crack generation and further propagation.
(2) The greatest flexural and compressive strength of CFRP composites was yielded by 2 wt.% PAN fiber, achieving 685.61 MPa and 286.66 MPa, respectively, and exhibiting 28.6% and 11.7% increments over the base strength.
(3) The introduction of PAN fiber contributed to shifting the failure modes of the CFRP composites from delamination-dominated failure to crossing-multi-layer failure.

In all, PAN fiber has been proven as a useful fiber that was used to improve the mechanical properties of CFRP composites, and this interleaving method could provide a better alternative for the development of high-performance FRP composites.

## Figures and Tables

**Table 1:** Physical properties and manufacturer summary of raw materials.

Raw Materials	Physical Properties	Manufacturer
Carbon fiber fabric	Density 1.76 g/cm^3^, tensile strength 3530 MPa and modulus 230 GPa	Shanghai Longchi Construction Technology Co., Ltd., Shanghai, China
PAN fiber	Density 1.18 g/cm^3^, tensile strength 800 MPa, modulus 16 GPa, and melting point 220–260 °C	Zibo Qiaotu Engineering Materials Co., Ltd., Shandong, China
Epoxy resin	105 Epoxy Resin, toxic (boiling point higher than 204 °C)	West System, bay city, MI, USA
Hardener	206 slow hardener, colorless or light yellow transparent liquid, toxic	West System, bay city, MI, USA

**Table 2:** Detailed information of CFRP composites with various conditions.

Specimen Group	PAN Fiber Mass Proportion (wt.%) in Mixture	Additive Amount in Each Interlayer (g/m^2^)	Thickness (mm)	Thickness Increment of Each Interlayer (μm)
Plain CFRP	0	0	1.96	0
1-PAN/CFRP	1	1.42	2	4.4
2-PAN/CFRP	2	2.84	1.99	3.3
3-PAN/CFRP	3	4.26	2.02	6.6
4-PAN/CFRP	4	5.7	2.04	8.8

**Table 3:** Detailed flexural and compressive test results of laminated CFRP composites with various mass proportions of PAN fiber at interlayer.

Specimens		Plain CFRP	1-PAN/CFRP	2-PAN/CFRP	3-PAN/CFRP	4-PAN/CFRP
PAN fiber mass proportion (wt.%)		0	1	2	3	4
Flexural strength (MPa)	Average	532.76	592.65	685.61	592.76	527.57
	Standard derivation	23.25	9.60	29.17	21.19	16.73
Energy absorption (J)	Average	0.61	0.67	0.74	0.63	0.53
	Standard derivation	0.05	0.04	0.05	0.08	0.05
Compressive strength (MPa)	Average	256.46	250.04	286.66	256.79	270.98
	Standard derivation	40.47	35.41	35.01	28.77	24.21
Energy absorption (J)	Average	3.06	3.1	3.4	2.82	3.34
	Standard derivation	1.03	0.61	1.54	0.57	1.08

## Data Availability

The original contributions presented in this study are included in the article. Further inquiries can be directed to the corresponding author(s).
